# Football sports automatic judgment model based on improved YOLOv7 and RNN

**DOI:** 10.1371/journal.pone.0334158

**Published:** 2025-11-05

**Authors:** Ting Wang, Xiao Yan, Jiawei Li, Xilong Luo

**Affiliations:** Physical Education College, Shanxi University, Taiyuan, China; Wuhan University of Science and Technology, CHINA

## Abstract

The extraction, classification, and judgment of sports video scenes can improve work efficiency and accuracy. To understand sports videos in dynamic scenes, this study applies deep learning technology, firstly introducing clustering algorithm and attention mechanism to improve the target detection technology You Only Look Once v7, and identifying the targets existing in the scene. Then, the sparrow search algorithm in artificial intelligence algorithm is taken to optimize the parameter search of the recurrent neural network and automatically extract the target scene. After introducing three optimization strategies, the proposed model achieved a detection accuracy of 0.993 (as measured by classification accuracy), a floating-point calculation times of 244, and a detection speed of 264.245 fps. The average detection accuracy of this model was 0.95, and the loss function curve converged with the minimum number of iterations and convergence value. The maximum correlation accuracy was 0.958, and the detection accuracy was 0.926. Meanwhile, the model had the highest intersection over union ratio and recall rate on different datasets, reaching 0.885 and 0.961 respectively on the TrackingNet dataset. The improved scene extraction model had the smallest three error values, with the highest accuracy of 0.932, F1 of 0.955, and subject working characteristic curve area of 0.969. The R-squared value and semantic consistency of scene extraction perform well, improving the accuracy and fairness of football sports judgment. This study proposes an innovative solution to address sports video scene recognition, improving the accuracy of sports video scene recognition and bringing new effective technological means to the field of sports video analysis. Meanwhile, this study contributes to the rapid development of the sports industry and promotes the automation and popularization of football.

## Introduction

Scene recognition is a key branch of computer vision and artificial intelligence, which can convert and classify unstructured data such as images and videos, which is applied in video surveillance and virtual reality. Video scene classification can divide video content into different semantic categories or scenes, relying on computer technology to achieve deep understanding and analysis of video content [1–2]. At present, the application of video scene classification in sports is becoming increasingly widespread. Classifying different actions and scenes in sports videos can help coaches and athletes quickly locate key scenes and events in the video, accurately identify target objects and motion patterns in the video. Video scene classification is beneficial for improving training efficiency, optimizing game analysis, and helping coaches evaluate athletes’ skill levels [3]. Meanwhile, dynamic video scene classification technology can process and analyze sports videos in real time, automatically judge sports matches, effectively reduce misjudgments and missed judgments. The automatic recognition technology for sports video scenes is to identify specific target objects from video frames, extract key scenes from the video, and ultimately automatically judge motions. Scene recognition involves the fusion and application of multiple key technologies, including object detection, object scene extraction, and related image processing, video analysis, and other technologies [4]. The existing dynamic scene recognition technology is facing the challenge of environmental light sources. Different lighting conditions may result in poor target recognition performance. The similar athlete postures, severe occlusion, and overlapping phenomena in sports videos seriously interfere with the recognition effect of the scene. The current scene recognition often ignores the temporal and spatial relationship of the target, which leads to its inability to be applied to dynamic sports video analysis. Therefore, to improve the scene recognition accuracy, adapt to complex scenes, and meet the demand for dynamic scene recognition in sports video analysis, it is necessary to carry out research on dynamic scene recognition models [5].

In other domains, similar challenges are being tackled with domain-specific solutions. For instance, Wu et al. proposed a lightweight MobileNetV2_DeepLabV3 segmentation network to measure dam crack width under complex lighting and structural conditions, highlighting the need of accurately detecting fine-grained targets in noisy and changing environments [6]. In agricultural robotics, Wu et al. enhanced CycleGAN to improve nighttime pineapple detection by addressing low-light visibility and dataset limitations, leading to significant improvements in precision and recall when integrated with You Only Look Once v7 (YOLOv7)-based detectors [7]. These examples emphasize that object detection in real-world scenarios often faces issues such as dynamic lighting, small object scale, and domain-specific constraints. Similarly, in sports video analysis, especially football match scenarios, these challenges are compounded by rapid motion, player occlusion, and unstructured spatial dynamics, which require more robust and adaptive spatio-temporal detection frameworks, such as the method proposed in this study.

To accurately analyze sports videos and address the challenges of current automatic recognition technologies in sports scenarios, this study takes football as an example and constructs a YOLOv7 model to solve the difficulty of scene recognition in sports videos. In addition, a target scene extraction model is designed using a recurrent neural network (RNN). The core research problem lies in accurately identifying actions related to key judgments in sports videos, such as dribbling, ball contact, and goalkeeper saves, especially in situations involving small targets, rapid movements, and high scene complexity to achieve high-precision detection and time recognition of actions.

The contribution of this study lies in constructing a multi-stage recognition framework that integrates an improved YOLOv7 detection module with a BiLSTM-SSA-based temporal recognition module. The detection component incorporates GhostConv to achieve lightweight structure, CBAM attention mechanism to enhance feature representation, and K-means clustering to optimize anchor boxes, thereby improving localization accuracy and adaptability. The recognition component utilizes BiLSTM to model action sequences and employs the Sparrow Search Algorithm to optimize key hyperparameters. Additionally, a dataset containing multiple types of typical referee-related actions is constructed, and action detection and recognition experiments area conducted based on this framework.

This study is structured into four distinct sections. The first section reviews the current research on object detection, scene recognition, and classification. The second section designs an improved Small Object Detection Algorithm (SODA) and target scene extraction model. The third section conducts performance testing and application analysis on the object detection and scene extraction model. The fourth section outlines the main conclusions of the experiment and future work.

### Related works

In recent years, with the rapid development of artificial intelligence and computer vision technologies, key action recognition and event detection in sports videos have become vital directions for intelligent analysis. In football videos, accurately recognizing technical actions such as dribbling, shooting, and defending is of great significance for tactical analysis and instructional applications. Researchers have proposed various technical approaches for sports video action recognition, covering deep learning, temporal modeling, and multi-modal fusion. X. Sun et al. proposed an action prediction framework that integrated LSTM and GAN, which improved the ability to predict action intentions in advance and solved the challenge of early action recognition in tennis. This provided technical insights for motion prediction in fast-paced sports such as football [8]. S. B. Khobdeh et al. focused on the complexity of basketball action recognition and proposed a model combining YOLO and deep fuzzy LSTM to achieve accurate recognition in complex backgrounds. The approach to target detection and temporal modeling was transferable to multi-target analysis in football videos [9]. M. A. Khan et al. tackled human action recognition in video surveillance by fusing multi-view and deep features, selecting key features using entropy and correlation metrics, and achieving outstanding performance across multiple datasets. The work offered inspiration for integrating multi-source information in multi-angle football videos [10]. In terms of temporal analysis, M. A. Sarwar et al. proposed a skeleton-based badminton keyframe detection mechanism that effectively decomposes macro level actions, providing a reference for identifying stage-based actions such as tackles and shots in football [11]. M. B. Shaikh et al. explored multi-modal fusion by developing a deep recognition framework combining audio-image and video data. The promising results on UCF51 and Kinetics datasets provided a new perspective for joint “action + speech” recognition in football matches [12].

In football-specific research, Z. Chen et al. proposed a children’s football action recognition method based on LSTM and V-DBN, which combined skeleton modeling and deep feature fusion, significantly improving the recognition accuracy of young athletes [13]. J. Wang et al. combined machine vision and IoT technologies to extract and recognize offensive action features in football players. A three-channel fusion model that combined RGB and depth information was proposed to enhance tactical action detection efficiency [14]. L. Xuan et al. designed a football action recognition algorithm based on deformable convolutional neural networks. By integrating binocular vision preprocessing and SVM classifiers, the model achieved superior performance in both recognition accuracy and frame rate stability [15]. P. Yang et al. introduced a deep learning-based action recognition method for football teaching videos by constructing the DL-FTMR model. This model fused inertial measurement unit data and image information to achieve high-precision automatic classification in instructional contexts, offering tool support for match training and skill assessment [16]. X. Zhao developed a football shooting action recognition method based on Bayesian classification. A Gaussian mixture model was taken to extract key features of shooting actions, striking a good balance between real-time performance and accuracy [17].

In summary, existing research has made significant progress in improving recognition accuracy, designing efficient model structures, and integrating multi-modal information in sports action recognition. Especially in football video analysis, researchers have innovated in detection accuracy, time modeling, and teaching applications. However, challenges such as weak model generalization, difficulty in recognizing small targets, and limited adaptability to complex backgrounds remain. To address these issues, this study proposes a multi-stage recognition framework that integrates an improved YOLOv7 detection module with a BiLSTM-SSA temporal recognition module, aiming to tackle the detection and recognition difficulties caused by rapid action changes, small object sizes, and high scene complexity in football videos. The innovation of the research lies in that for the first time, it proposes a multi-stage recognition strategy integrating improved YOLOv7 and RNN to address issues such as small targets, rapid changes in actions, and complex interweaving of backgrounds in football videos. Technically, the innovation lies in deeply embedding the GhostConv and CBAM mechanisms into the detection network, enhancing the detection efficiency in resource-constrained scenarios and its sensitivity to key action areas. Meanwhile, the SSA algorithm is applied to hyperparameter tuning in the time series recognition module, which significantly improves the training stability and generalization ability of the model while maintaining recognition accuracy.

### Design of an automatic soccer motion judgment model based on object detection and scene extraction

To solve the recognition problem of football sports scenes and face complex sports field environments, a SODA is first designed with YOLOv7. Then, an improved RNN visual scene extraction model is proposed.

### Soda with enhanced YOLOv7

**Fig 1:**
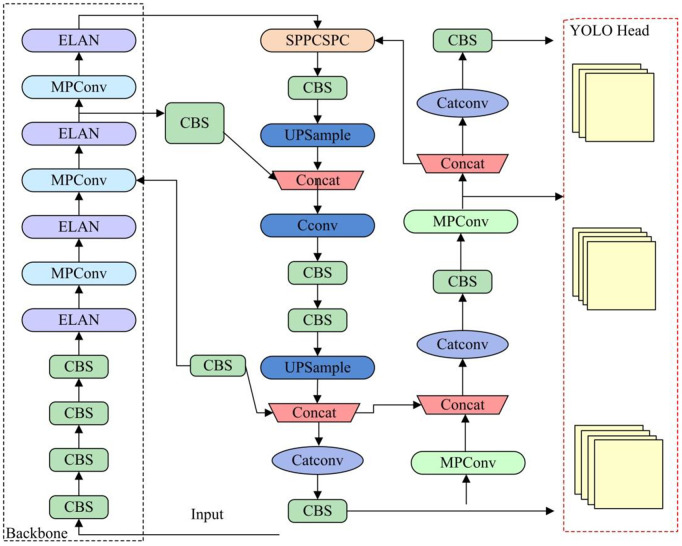
YOLOv7 network structure composition diagram.

SODA is a fundamental step in sports video scene recognition, aimed at identifying specific target objects from video frames [18–20]. This study selects the object detection model YOLOv7 to design a small object detection model for football sports. The YOLOv7 network is improved from the YOLO series algorithms, mainly including input layer, backbone network, Neck layer, and output terminal. The structural composition is shown in Fig 1 [21–24].

**Fig 2:**
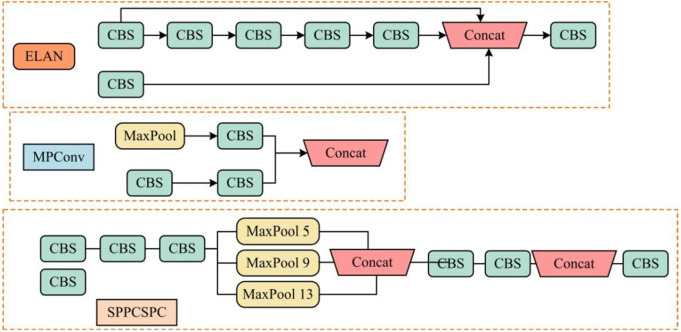
Schematic diagram of ELAN module, MPConv, and SPPCSPC module composition.

The input layer is mainly responsible for receiving and processing images, adjusting the input image to a fixed size of 640 × 640 for subsequent processing [25–27]. Subsequently, four images are loaded, and the Mosaic data augmentation method is used to segment, combine, and change the position of the images, increasing the diversity and richness of the training dataset. The backbone network of YOLOv7 includes a feature extraction module, that is, a Convolutional Batch Normalization and Silu Activation Layer (CBS), an Efficient Layer Aggregation Network (ELAN), an MPConv (MaxPool and Convolutional) module, and a Spatial Pyramid Pooling Convolutional Neural Network (SPPCSPC). The structural composition of the backbone network is shown in Fig 2 [28–30].

In Fig 2, the ELAN serves to control the length and diversity of gradient paths, including two branches. The MPConv module combines MaxPool and convolution operations, down-sampling through two branches. The feature fusion improves the generalization ability of the network [31–32]. The SPPCSPC module combines Spatial Pyramid Pooling (SPP) and Cross-Stage Partial Connection (CSP) modules. SPP extracts multi-scale features through pooling operations at different scales, while CSP optimizes the feature extraction process through cross-stage partial connections [33]. The CBS module uses the SiLU activation function, as shown in Equation (1).


SiLU(x)=x·sigmoid(x) (1)

In Equation (1), x denotes the input data. The batch normalization layer of the CBS module is responsible for accelerating training and providing model stability. The calculation process is shown in Equation (2).


$$y^{\left(k\right)}=\gamma^{\left(k\right)}\frac{x^{\left(k\right)}-\mu^{\left(k\right)}}{\sqrt{{\left(\sigma^{\left(k\right)}\right)}^{\text{2}}+\varepsilon }}+\beta^{\left(k\right)}$$ (2)

In Equation (2), $x^{\left(k\right)}$ and $y^{\left(k\right)}$ denote input and output data, respectively. $\mu^{\left(k\right)}$ and $\sigma^{\left(k\right)}$ represent the mean and standard deviation. $x^{\left(k\right)}$, $\gamma^{\left(k\right)}$, and $\beta^{\left(k\right)}$ represent translation parameters and scaling parameters, respectively. k and ε represent dimensions and constants respectively. The Backbone layer uses the HardSwish activation function, as expressed in Equation (3).


$$f(x)=x\times \frac{ReLU6(x+3)}{6}$$ (3)

**Fig 3:**
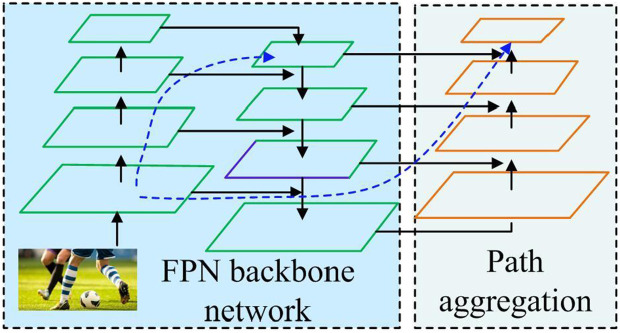
Schematic diagram of neck layer FPN and APN structure.

In Fig 3, the Neck layer of YOLOv7 adopts the traditional Path Aggregation Network (PAN)-Feature Pyramid Network (FPN) structure.

In Fig 3, PAN-FPN fuses features of different scales, conveying feature information from top to bottom and high-level features from bottom to top. The system integrates data from disparate sources and scales, thereby enhancing the efficacy of the detection process [34]. The Head layer of YOLOv7 is the part that outputs the detection results, including the detection head and the re-parameterized convolution RepConv module. The detection head receives fused features from the neck network and processing the output target information through convolution and activation functions [35]. The RepConv module adjusts the number of channels, first merging the convolutional layer and normalization layer to achieve parameter fusion, and then determining the maximum scale convolution. The confidence level of the bounding box ζ and the intersection over union ratio IoU are predicted, as shown in Equation (4).


$$\left\{ \begin{array}{c} \zeta =\mathrm{Pr}\left(object\right)*IoU_{pred}^{teuth}\\ IoU\left(\hat{b},b\right)=\frac{\left|\hat{b}\cap b\right|}{\left|\hat{b}\cup b\right|} \end{array}\right.$$ (4)

In Equation (4), Pr(object) represents the probability of the bounding box containing the detection target. $\hat{b}$ and b represent the predicted and true bounding boxes, respectively. Equation (5) shows the binary cross-entropy loss function L.


$$L=-\sum_{n=1}^{N}y_{i}^{*}\log\left(y_{i}\right)+\left(1-y_{i}^{*}\right)\log\left(1-y_{i}\right)$$ (5)

In Equation (5), $y_{i}^{*}$ represents the true value of the sample. N is the quantity of samples. $y_{i}$ is the model output. In the traditional YOLOv7 training process, the central coordinates of the predicted bounding box are determined by Equation (6).


$$\left\{ \begin{array}{c} b_{x}=\sigma \left(t_{x}\right)+c_{x}\\ b_{y}=\sigma \left(t_{y}\right)+c_{y}\\ b_{h}=P_{h}\times e^{t_{h}}\\ b_{w}=P_{w}\times e^{t_{w}} \end{array}\right.$$ (6)

In Equation (6), $\sigma \left(t_{x}\right)$ and $\sigma \left(t_{y}\right)$ respectively represent the displacement offset of the anchor box center point relative to the upper left corner of the detection grid. $c_{x}$ and $c_{y}$ respectively represent the relative displacement coordinates of the anchor box on the detection grid. $P_{h}$ and $P_{w}$ represent the width and height of the initial anchor box. $t_{h}$ and $t_{w}$ represent the width and height scaling of the anchor box. $b_{x}$, $b_{y}$, $b_{h}$, and $b_{w}$ respectively denote the horizontal, vertical, height, and width of the prediction box.

The distribution, size change, and movement pattern of key elements in the screen such as football, player, and goal in the football movement judgement task affect the determination of the bounding box, and consideration should be given to designing an initialised anchor box that is more in line with the characteristics of the football movement. However, there is usually a significant difference in the size between the initialization bounding box and the detection target, which can easily lead to missed or false detections. Therefore, this study optimizes the built-in anchor box of YOLOv7. The study takes the K-means to first cluster the shape, size, and dimensions of sample targets. Based on the K-means results, the adjustment process of bounding boxes is simplified to reduce training losses. The clustering objective function is calculated by Equation (7).


$$J\left(X,\pi \right)={\sum_{j=1}^{k}\sum_{i\in \pi_{j}}\backslash |x_{i}-m_{j}\backslash |}^{\text{2}}$$ (7)

In Equation (7), $\pi_{j}$ represents the class j sample. $x_{i}$ represents the data point. $m_{j}$ represents the center of the class j sample. To ensure that the anchor boxes better represent actual object sizes, a total of 12,000 labeled bounding boxes are used for K-means clustering, extracted from approximately 4,000 annotated image frames in the training dataset. The number of clusters K is set to 9, following common practice in YOLO-based architectures, which balances small, medium, and large object detection. This sampling strategy enhances the robustness and generalizability of anchor box initialization.

In summary, the improved YOLOv7 adopts the K-means clustering algorithm to cluster the sample labeled bounding box sizes, which can generate initialized anchor frames that are closer to the actual target sizes. Compared with traditional anchor frames that come with the YOLOv7, they have stronger adaptability and accuracy. Making anchor frames more representative of the target size in the dataset greatly reduces the difficulty of bounding box adjustment, which is beneficial for improving the detection accuracy of small targets in sports videos.

**Fig 4:**
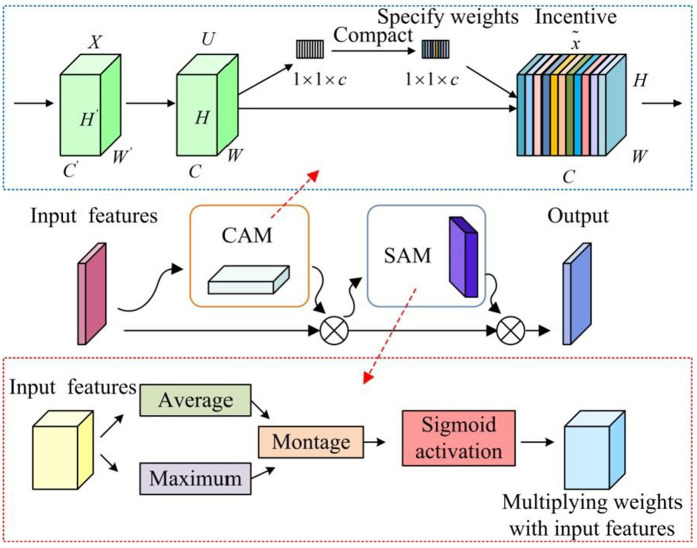
Schematic diagram of the structure components of CBAM.

Capturing key actions such as player interactions, ball transfers and shots on goal, considering player positional relationships, team tactical layouts, etc. is critical to understanding the actions and events. In Fig 4, to further enhance the attention to important features in the detection process, the research introduces the Convolutional Block Attention Module (CBAM) in the attention mechanism.

In Fig 4, CBAM includes Channel Attention Mechanism (CAM) and Spatial Attention Mechanism (SAM). CAM utilizes the compression module to complete feature learning and information compression, and uses the excitation module to allocate channel weights [36]. The implementation process of CAM is shown in Equation (8).


$$M_{c}(F)=\sigma \left(MLP\left(AvgPool(F)\right)+MLP\left(MaxPool(F)\right)\right)$$ (8)

In Equation (8), $M_{c}$ represents channel attention. AvgPool and MaxPool are average pooling and maximum pooling. MLP is a multi-layer perceptron. σ is an activation function. F represents input image features. Equation (9) shows the implementation process of SAM.


$$M_{s}(F)=\sigma \left(f^{7\times 7}\left[\left(AvgPool(F)\right);\left(MaxPool(F)\right)\right]\right)$$ (9)

In Equation (9), $M_{s}$ is the spatial attention. $f^{7\times 7}$ is the convolution operation. The introduction of the attention mechanism module enables the model to pay more attention to the feature information that is crucial for target detection by dynamically adjusting the network weights, thus reducing the redundant learning of background information and improving the generalization ability and robustness. Compared with the traditional YOLOv7, it is prone to different interference when dealing with situations such as complex background and target occlusion, which affects the accuracy of the model.

Finally, the GhostConv convolution layer is introduced at the beginning of the ELAN module for lightweight improvement. For GhostConv, a limited number of convolutional kernels are employed to extract pertinent features, then a more cost-effective linear transformation operation is used.

### Sports scene extraction model based on improved RNN

Target scene extraction is the process of further extracting key scenes from videos based on object detection, involving techniques such as scene segmentation, event recognition, and spatio-temporal information fusion. After the scene units are divided, key scenes are identified and extracted by analyzing the target objects, actions, and contextual information in the scene. Dynamic video scene classification considers spatial information such as target position and features in each frame of the image. Time information, i.e. the behavior or changes of the target between different frames, also needs to be considered. In this study, “shooting” in football is taken as the target scene analysis object, and a video frame feature representation method is designed based on one hot feature representation. A vector containing spatial information is used to represent the feature target, which is the coordinate information of the regression box obtained from the improved YOLOv7. Meanwhile, this study designs five judgment labels for the “shooting” scenario. The first label represents the football running on the field. The second label represents the athlete dribbling. The third label represents the football rubbing against the outside of the door frame and leaving the bottom line. The fourth label represents the football being saved by the goalkeeper. The fifth label represents the football being hit and entering the goal. Motion behaviour in football games usually involves a series of consecutive actions, and the actions have specific laws and patterns in the time series. For the football shooting scene, the research can delve into the time series analysis technique to better capture and identify the shooting behaviour. Therefore, RNN is selected for the target scene extraction model design.

**Fig 5:**
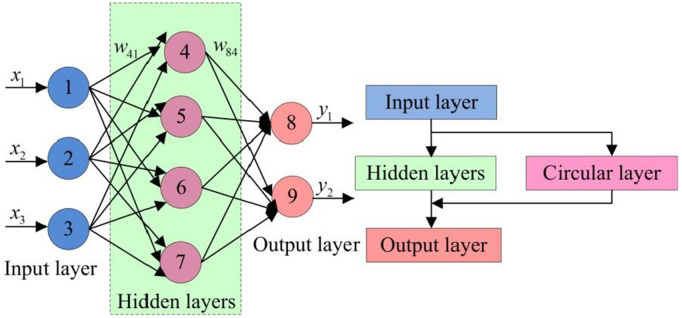
Schematic diagram of RNNs.

RNN is mainly used for processing and predicting patterns in sequence data. Unlike traditional feedforward neural networks, RNN is more suitable for handling temporal dependencies or temporal dynamics in sequence data. It is suitable for designing video dynamic scene extraction models [37–38]. RNN has a “memory function” that can perform the same task on elements in a sequence and output historical calculations that depend on the sequence, as shown in Fig 5.

In Fig 5, the nodes between the hidden layers of the RNN are interconnected to determine the output at the next moment. The hidden state is shown in Equation (10).


$$\left\{ \begin{array}{c} h(t)=ox\left(t-1\right)+Ws\left(t-1\right)\\ s(t)=f\left(h(t)\right)\\ o(t)=g\left(Ws(t)\right) \end{array}\right.$$ (10)

In Equation (10), t and t−1 denote different time series. s(t) represents the memory. f() denotes the activation function of tanh. g() is the softmax activation function. W represents the weight matrix. o represents the output). Traditional RNN suffers from gradient vanishing or exploding problems when dealing with sequences of arbitrary length, making it difficult for RNN to capture useful information between the beginning and end of the sequence. The study adopts a variant structure of RNN - Bidirectional Long Short Term Memory (BiLSTM) network. BiLSTM introduces a bidirectional processing mechanism into LSTM, expanding its ability to understand context in sequence prediction tasks [39–40]. The LSTM unit consists of a cell state and three “gate” structures, as shown in Equation (11).


$$\left\{ \begin{array}{c} i_{t}=\sigma \left(W_{i}\left[y_{t-1},x_{t}\right]+b_{i}\right)\\ \begin{array}{c} O_{t}=\sigma \left(W_{o}\left[y_{t-1},x_{t}\right]+b_{o}\right)\\ f_{t}=\sigma \left(W_{f}\left[y_{t-1},x_{t}\right]+b_{f}\right) \end{array} \end{array}\right.$$ (11)

**Fig 6:**
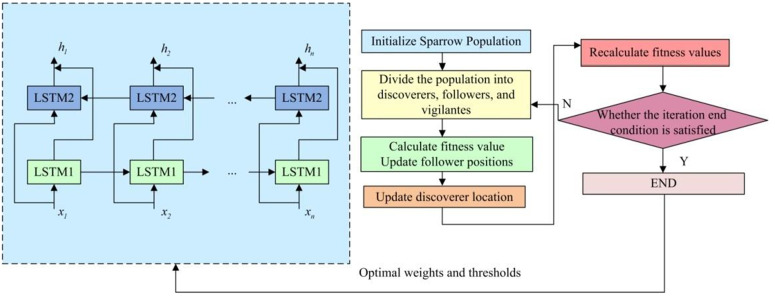
Schematic diagram of the improved RNNs structure.

In Equation (11), b represents the bias. After obtaining the scene extraction model based on BiLSTM, this study defines the scene before the football leaves the baseline, before entering the goal, or when the player dribbles the ball last as the start frame of the video. The end frame of the video is defined as the football rubbing the outside of the door frame beyond the baseline, the football being saved by the goalkeeper, or entering the goal. However, in the process of extracting target scenes, the traditional BiLSTM model is prone to misjudging video frames due to the switching of shooting shots, making it difficult to distinguish ownership relationships between players. In addition, the appearance of “high-altitude balls” is prone to misjudgment, which can easily lead to the end frame of the video. Therefore, this study conducts an improvement analysis of BiLSTM. Due to the heavy dependence of BiLSTM on initial thresholds, weights, and other hyperparameters, the Sparrow Search Algorithm (SSA) is introduced for hyperparameter optimization, as shown in Fig 6.

In Fig 6, SSA is responsible for optimizing the weights and initial thresholds. SSA has good performance and applicability, and can improve the accuracy of BILSTM in scene extraction. In the sparrow population, the discoverers finds food and provides direction and area for the entire sparrow population to forage. The updated location $X_{i,j}^{t+1}$ is shown in Equation (12).


$$X_{i,j}^{t^{\prime }+1}=\left\{ \begin{array}{c} X_{i,j}^{t^{\prime }}\cdot \exp\left(\frac{-i}{\alpha \cdot iter_{\max}}\right)\;\;\;\;if\;R_{\text{2}}<ST\\ X_{i,j}^{t^{\prime }}+Q\cdot L^{\prime }\;\;\;\;\;\;if\;R_{\text{2}}\geq ST \end{array}\right.$$ (12)

In Equation (12), $t^{\prime }$ represents the number of iterations. $iter_{\max}$ is the largest iteration. i and j denote the number of sparrows and their location dimensions, respectively. α and Q represent random constants. $R_{\text{2}}$ and ST represent alarm values and safety thresholds, respectively. $L^{\prime }$ represents the d -dimensional matrix. Followers use discoverers to obtain food, and location updates are shown in Equation (13).


$$X_{i,j}^{t^{\prime }+1}=\left\{ \begin{array}{c} Q\cdot \exp\left(\frac{x_{worst}^{t^{\prime }}-x_{i,j}^{t^{\prime }}}{i^{\text{2}}}\right)\;\;\;\;if\;i>n/2\\ X_{p}^{t^{\prime }+1}+\left|X_{i,j}^{t^{\prime }}-X_{p}^{t^{\prime }+1}\right|\cdot A^{+}\cdot L^{\prime }\;\;\;\;\;if\;R_{\text{2}}\leq n/2 \end{array}\right.$$ (13)

In Equation (13), $X_{p}$ and $X_{worst}$ are the best and worst positions of the discoverer. A represents the matrix of 1×d. The process of updating the position of the warning agent is shown in Equation (14).


$$X_{i,j}^{t^{\prime }+1}=\left\{ \begin{array}{c} Q\cdot \exp\left(\frac{x_{worst}^{t^{\prime }}-x_{i,j}^{t^{\prime }}}{i^{\text{2}}}\right)\;\;\;\;if\;i>n/2\\ X_{p}^{t^{\prime }+1}+\left|X_{i,j}^{t^{\prime }}-X_{p}^{t^{\prime }+1}\right|\cdot A^{+}\cdot L^{\prime }\;\;\;\;\;if\;R_{\text{2}}\leq n/2 \end{array}\right.$$ (14)

**Fig 7:**
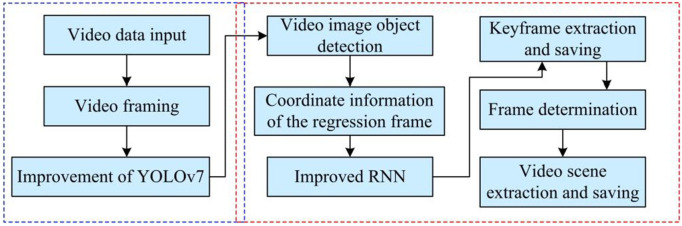
Schematic diagram of automatic judgment model architecture for football sports.

In Equation (14), β and K are constants that adjust the step size and the direction of the population. $f_{i}$, $f_{w}$, and $f_{g}$ respectively represent the fitness value, optimal fitness value, and worst fitness value of the population. ε represents the minimum constant. Finally, the architecture of the football motion automatic judgment model based on improved YOLOv7 and improved RNN is shown in Fig 7.

In Fig 7, the model architecture mainly consists of two parts. After completing the input and frame processing of video data, the improved YOLOv7 and improved RNN are used to perform object detection and video scene extraction in sequence. Before the video scene, the video frame determination needs to be completed. The improved YOLOv7 model is responsible for recognizing footballs and athletes in video frames and obtaining their positional information, i.e. the coordinates of the regression frames. Then, the target positions and features are used as inputs to the improved RNN, which is used to process the time series data, i.e., to analyse the behaviors or changes of the target objects in the consecutive video frames in order to identify the shooting scenarios. Finally, based on the output of the RNN model, it determines whether the current video frame belongs to the shooting scene or not, and outputs the corresponding decision labels.

### Performance analysis of automatic judgment model for football sports

To test the model for football sports videos, this study conducted tests on detection performance and judgment application effects, and discussed the results.

### Performance testing of improving YOLOv7 soda

The deep learning framework adopted in this study is PyTorch. The operating platforms include the Intel Core i7-11700@2.5GHz processor, the NVIDIA GeForce GTX 1650 graphics card (4.0GB video memory), and 64.0GB of memory. When evaluating the operational efficiency of the model (such as FPS), the image input resolution is uniformly set to 1,280 × 720, the batch size is set to 1, and the original video frames are taken as the processing unit. The test videos are all selected from the actual competition scenes and have complex features such as multiple targets, high dynamics, and occlusion interference. It ensures the authenticity and challenge of the experimental evaluation. The experiment selects VisDrone2019, OTB100, GOT-10k, and TrackingNet as the experimental datasets. VisDrone2019 contains a large amount of image and video data, mainly used for small object recognition and detection. The OTB100 dataset contains 100 video sequences, each containing a specific tracked target and scene change. The GOT-10k dataset contains over 10,000 video frames, covering various complex environments and scenes. TrackingNet contains 30,643 video clips, with an average duration of 16.6 seconds per video clip.

To verify the effects of CBAM, GhostConv module, and K-means on SODA, the ablation experiment is first conducted. The comparative models include Single Shot Multibox Detector (SSD) and Faster Region Convolutional Neural Network (Faster R-CNN). In Table 1, the improved YOLOv7 had the highest value in floating-point calculation times, Detection Speed (fps), and detection accuracy, indicating the best model performance. The CBAM module increased the floating-point calculation times by 8 flops, detection speed by 7.68 fps, and detection accuracy by 0.149 for the improved YOLOv7. After introducing the GhostConv module, the floating-point calculation times and detection speed of the improved YOLOv7 were increased to 194 flops and 159.104fps, respectively, with a relatively small increase in detection accuracy of only 0.011. The lightweight design of GhostConv module mainly improved the calculation speed and computing ability. After introducing K-means, the detection accuracy of the YOLOv7 was improved to 0.993, reaching the optimal level. Meanwhile, the number of floating-point calculations and detection speed reached 244 flops and 264.245 fps, respectively. Furthermore, to assess the reliability of these improvements, paired t-tests are performed on the key metrics. The results show that the improvements brought by CBAM and K-means are statistically significant at the p < 0.05 level, confirming that the observed gains are unlikely due to random variations.

**Table 1:** Comparison of ablation test results.

Method				Floating point calculation count (flops)	Speed (fps)	Accuracy
**YOLOv7**	**CBAM**	**GhostConv**	**K-means**			
√	/	/	/	113	128.967	0.745
√	√	/	/	121	136.647	0.894
√	√	√	/	194	159.104	0.905
√	√	√	√	244	264.245	0.993

**Fig 8:**
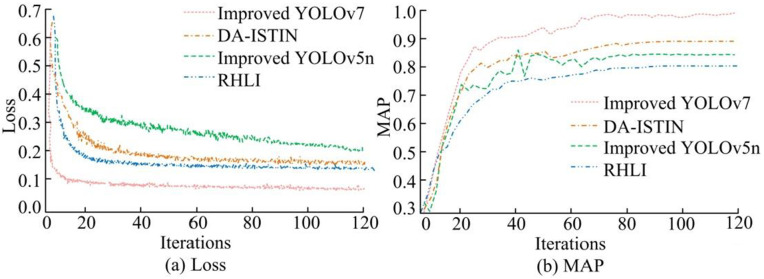
Comparison of average precision mean and loss function for different models.

The research method was compared with existing advanced models, including the DA-ISTIN that combines Double Attention (DA) model and Individual Spatial Temporal Inference Network (ISTIN) object detection model in reference [14], the Recurrent Historical Localization Information (RHLI) in reference [15], and the improved YOLOv5n in reference [19]. Fig 8 shows the Mean Average Precision (MAP) and loss function curves of different object detection models. In Fig 8 (a), the improved YOLOv7 converged with the minimum number of iterations, reached the convergence level at the beginning of the iteration, and had the minimum convergence value of 0.09. Compared to the DA-ISTIN, RHLI, and improved YOLOv5n, the improved YOLOv7 was reduced by 0.11, 0.08, and 0.14, respectively. The loss function curve could evaluate the changes in loss values. Overall, the research method demonstrated strong learning efficiency. In Fig 8(b), the MAP of the improved YOLOv7 reached 0.95 after 120 iterations. Under the same experimental environment, the MAP value of the DA-ISTIN was 0.85, the RHLI was 0.77, and the improved YOLOv5n was 0.81. Therefore, the YOLOv7 was improved using CBAM, GhostConv module, and K-means, resulting in improved detection accuracy and loss.

**Fig 9:**
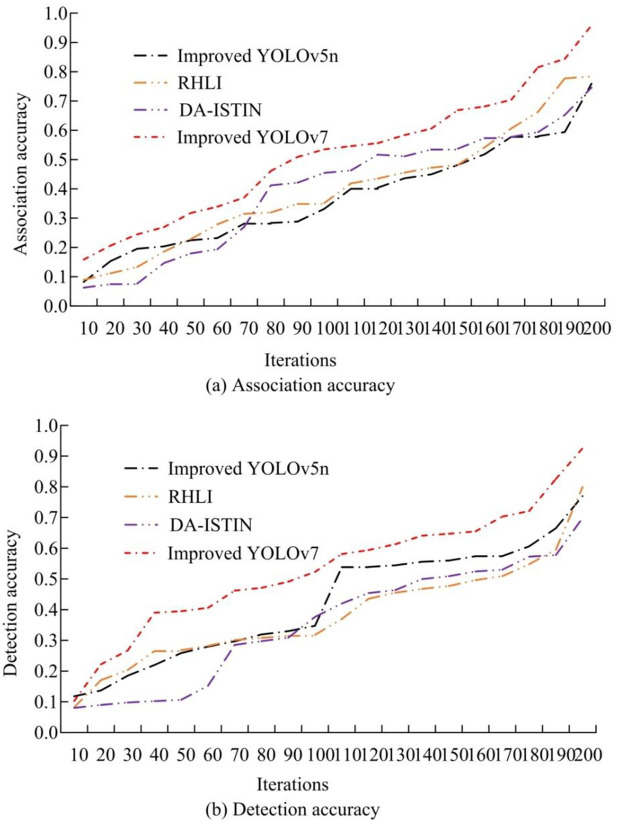
Results of association accuracy and detection accuracy of different models.

The association accuracy and detection accuracy metrics were applied. In Fig 9(a), the maximum AssA value of the improved YOLOv7 designed for research was 0.958, which was significantly improved. The association accuracy values of the DA-ISTIN, RHLI, and improved YOLOv5nwere 0.746, 0.784, and 0.759, respectively. In Fig 9(b), the improved YOLOv7’s maximum detection accuracy value was 0.926, while the association accuracy values of the DA-ISTIN, RHLI, and improved YOLOv5nwere 0.699, 0.799, and 0.770, respectively. Association accuracy could evaluate the accuracy of algorithms in associating targets. Overall, the improved YOLOv7 exhibited the best detection performance.

**Fig 10:**
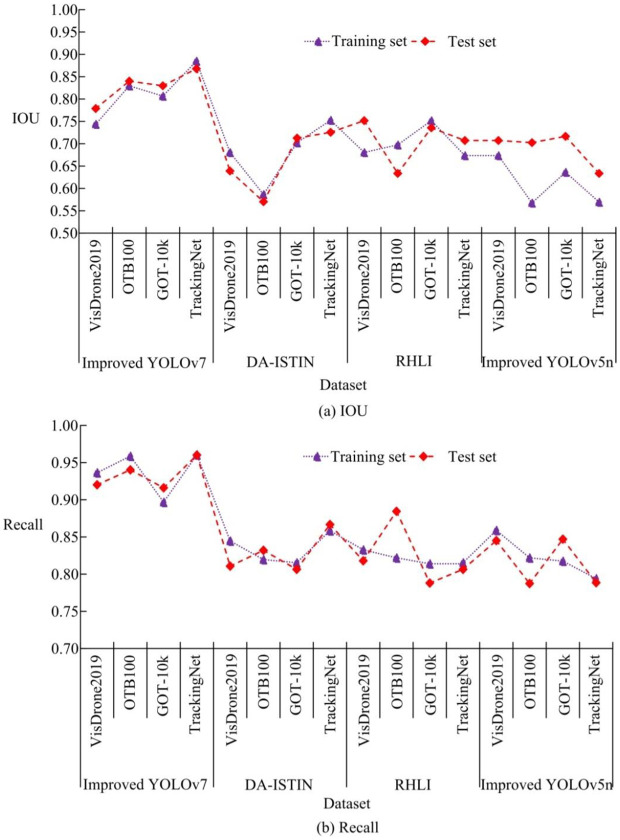
Comparison of IOU and recall of different detection models.

Different detection models are compared in terms of Intersection over Union (IOU) and recall. As shown in Fig 10(a), the IOU values varied significantly among the models. The improved YOLOv7 demonstrates consistently strong performance across both training and test sets, with the highest IOU reaching 0.885 on the TrackingNet dataset. In contrast, the highest IOU values for DA-ISTIN, RHLI, and improved YOLOv5n were 0.753, 0.752, and 0.717, respectively, showing a clear gap. Further analysis reveals that YOLOv7 not only achieves the highest peak performance, but also maintains smaller fluctuations, indicating better stability and robustness. In Fig 10(b), the recall rates were clearly differentiated. The improved YOLOv7 achieved a recall of 0.961 on the TrackingNet dataset, outperforming DA-ISTIN (0.806), RHLI (0.788), and improved YOLOv5n (0.788). Compared with DA-ISTIN, the improvement reached 19.16%, RHLI was 21.95%, and improved YOLOv5n was 22.05%. Although other models often suffer from missed detections or incomplete recognition in complex video scenes, the improved YOLOv7 can more comprehensively recognize targets, improve detection integrity, and demonstrate excellent object recovery and generalization capabilities. Furthermore, the limited recognition accuracy of DA-ISTIN and RHLI in complex video conditions can be attributed to their emphasis on spatio-temporal modeling, which lacks fine-grained extraction of object appearance features. These models often struggle with occlusions, motion blur, or distant targets, leading to tracking drift or missed detections. In contrast, the improved YOLOv7 benefits from the CBAM module, which enhances attention to key features, and from the K-means-based anchor box optimization, which improves adaptability to objects of various sizes. As a result, it can consistently produce accurate bounding boxes even in scenarios involving small objects or partial occlusions.

### Performance testing of improved RNN scene extraction model

Four high-definition football match videos are crawled for the research, covering different types of professional competitions, including intense midfield battles, attacking in front of the goal, defensive organization and corner kick positioning, etc. Representative video stream data are manually selected and four different data subsets are respectively constructed. The video frame segmentation is completed using the Ffmpeg tool. A total of 13,464 frames of image data are obtained. The total duration of the video is approximately 165 minutes, the frame rate is uniformly 30fps, and the average length of each video segment is approximately 6.8 minutes. The video shooting source is a rebroadcast-level fixed dual-camera perspective. The cameras are installed at both ends of the stadium, with full coverage and some close-up shots, capable of capturing small targets, high-speed movements, and blocking movements. A total of six key football action scenarios are labeled in the dataset, namely dribbling and breakthrough, passing, shooting, defense, saves, and fouls. The sample distribution among the categories remains relatively balanced.

To ensure the quality and consistency of manual annotations, a dual-annotator and cross-validation strategy was adopted. Specifically, two researchers with experience in computer vision and sports action analysis independently labeled the video frames. A random subset of 2,000 frames was selected to calculate the agreement ratio, which reached 94.8%. Disagreements were resolved through adjudication by a third expert. The overall inter-rater reliability, measured by Cohen’s Kappa coefficient, was 0.923, indicating high annotation consistency.

**Fig 11:**
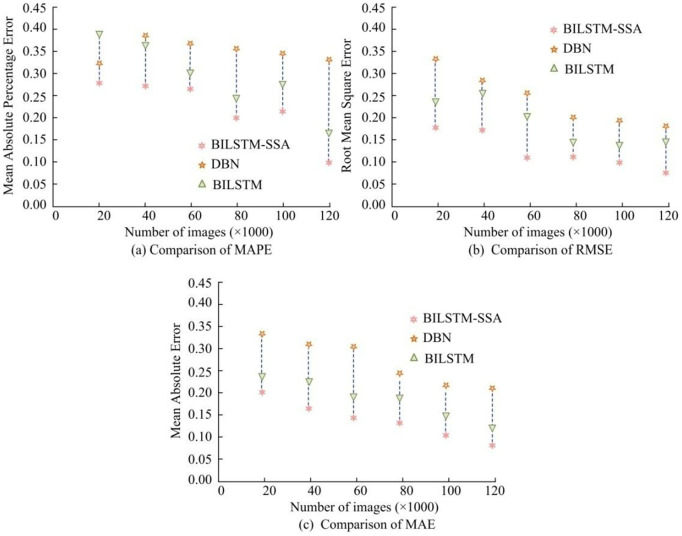
Scene extraction accuracy of different models.

The traditional BILSTM model and Deep Belief Network (DBN) are compared. Firstly, the accuracy of scene extraction by different models was evaluated, with Mean Absolute Percentage Error (MAPE), Root Mean Square Error (RMSE), and Mean Absolute Error (MAE) are compared. In Fig 11, the error index values of different models generally showed a downward trend. The MAPE, RMSE, and MAE of the BILSTM-SSA model under the same experimental environment were all at the lowest level, at 0.11, 0.09, and 0.08. The BILSTM-SSA model had significant advantages in scene extraction, and the hyperparameter optimization improved the the model’s ability to judge video frames.

This study introduced accuracy, Receiver Operating Characteristic Curve (ROC), and F1. In Table 2, the BILSTM-SSA model showed significant improvements in accuracy, Area Under Curve (AUC), and F1 compared to before the improvement. The BILSTM-SSA model had good stability performance on both the test and training sets, with a maximum accuracy of 0.932, F1 of 0.955, and AUC of 0.969. The improved BILSTM model was more accurate in scene extraction.

**Table 2:** Comparison of scene extraction capabilities of different models.

Model		Test set			Training set 113 128.967		
		Accuracy	F1	AUC	Accuracy	F1	AUC
**BILSTM-SSA**	**Video 1**	0.904	0.914	0.941	0.951	0.946	0.969
	**Video 2**	0.929	0.907	0.969	0.932	0.943	0.914
	**Video 3**	0.909	0.953	0.964	0.953	0.989	0.911
	**Video 4**	0.932	0.955	0.924	0.955	0.939	0.903
**BILSTM**	**Video 1**	0.882	0.891	0.882	0.874	0.870	0.874
	**Video 2**	0.893	0.855	0.893	0.860	0.852	0.860
	**Video 3**	0.876	0.877	0.876	0.859	0.869	0.859
	**Video 4**	0.849	0.849	0.849	0.897	0.849	0.897
**DBN**	**Video 1**	0.856	0.883	0.805	0.846	0.859	0.769
	**Video 2**	0.851	0.840	0.842	0.780	0.886	0.857
	**Video 3**	0.837	0.772	0.872	0.870	0.749	0.853
	**Video 4**	0.890	0.837	0.771	0.788	0.824	0.828

To evaluate the impact of SSA on the computational overhead in the BiLSTM optimization process, A comparative experiment was designed to test the impact of introducing SSA optimization mechanism on training time and inference speed under the same dataset and model structure. The experimental setup was as follows: The BILSTM model and the BILSTM-SSA model were trained respectively on four video data segments, and the total training time and the average inference time per frame were recorded. The experimental results are shown in Table 3. The SSA increased the training time by approximately 17.8%, but the frame processing time in the inference stage remained basically stable (with a difference of less than 3ms), indicating that the acceptable computational overhead does not affect the real-time application ability of the model in the actual penalty system.

**Table 3:** The impact of computational overhead on experimental results.

Model	Average training time (hrs)	Average inference time per frame (s)	Optimization method
**BiLSTM**	3.26	0.042	Manual
**BiLSTM-SSA**	3.84	0.045	SSA search
**Model**	Average training time (hrs)	Average inference time per frame (s)	Optimization method

**Fig 12:**
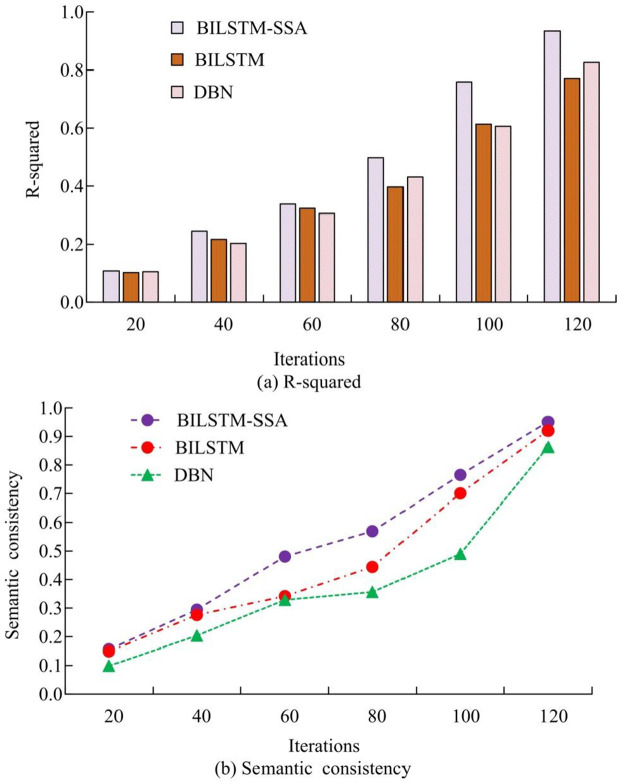
Comparison of semantic consistency and R-squared values of different models.

The semantic consistency and R-squared value of scene extraction by different models are compared. In Fig 12(a), the R-squared value of the BILSTM-SSA model reached its highest value of 0.934 as the iteration progressed. The R-squared values of the other two models were 0.772 and 0.826. The R-squared value measured the explanatory power of the model on the dependent variable, indicating that the BILSTM-SSA model could effectively utilize spatio-temporal data to fit features to accurately judge video frames. In Fig 12(b), the semantic consistency of the BILSTM-SSA model reached its highest value of 0.951 as the iteration process progressed. The semantic consistency values of the other two models were 0.920 and 0.863, respectively. Semantic consistency could evaluate the accuracy of the model in classifying image regions into correct semantic categories in scene analysis. The improved BILSTM model could effectively distinguish the semantic boundaries of different video frame images.

To further validate the generalization capability of the proposed model and ensure domain-specific performance evaluation, an additional set of supplementary experiments is conducted. Four publicly available and representative football video datasets are selected: Semantic Action Spotting (SoccerNet), Multi-View Temporal Detection (SoccerNet-V2), Fine-Grained Event Recognition (FootballNet), and Sports-Specific Multi-Object Tracking (SportsMOT). These datasets cover more football match scenarios, including various camera angles, event types, player interactions, and occlusion levels. For model comparison, three recent state-of-the-art methods are selected: RT-DETR, VID-Transformer, and TrackFormer. All models are evaluated under consistent data partitioning and metrics, including MAP, association accuracy, detection accuracy, Recall, and IOU. The results are shown in Table 4. The improved YOLOv7 combined with BILSTM-SSA achieved superior performance across all datasets. For instance, on the SoccerNet dataset, the proposed method achieved a MAP of 0.842 and a Recall of 0.851, significantly outperforming RT-DETR (MAP: 0.807) and VID-Transformer (MAP: 0.796). Consistent advantages were also observed on SoccerNet-V2 and SportsMOT, with excellent robustness maintained under the occlusion conditions of FootballNet. Overall, the proposed model demonstrates stronger stability and adaptability across various scenarios and outperforms existing methods in detection accuracy, scene recognition, and spatio-temporal feature modeling, verifying the effectiveness of the proposed method.

**Table 4:** Comparison of different models on public datasets.

Dataset	Method	MAP	Association accuracy	Detection accuracy	Recall	IOU
**SoccerNet**	**The proposed method**	0.842	0.818	0.806	0.851	0.793
	**RT-DETR**	0.807	0.786	0.774	0.811	0.762
	**VID-Transformer**	0.796	0.771	0.765	0.804	0.755
	**TrackFormer**	0.783	0.759	0.748	0.795	0.743
**SoccerNet-V2**	**The proposed method**	0.871	0.859	0.846	0.874	0.821
	**RT-DETR**	0.839	0.823	0.809	0.841	0.796
	**VID-Transformer**	0.824	0.811	0.797	0.833	0.781
	**TrackFormer**	0.812	0.799	0.784	0.820	0.771
**FootballNet**	**The proposed method**	0.816	0.801	0.789	0.814	0.777
	**RT-DETR**	0.782	0.768	0.751	0.791	0.748
	**VID-Transformer**	0.775	0.752	0.741	0.783	0.739
	**TrackFormer**	0.763	0.746	0.732	0.774	0.725
**SportsMOT**	**The proposed method**	0.862	0.841	0.828	0.879	0.812
	**RT-DETR**	0.831	0.809	0.793	0.846	0.787
	**VID-Transformer**	0.822	0.801	0.785	0.832	0.776
	**TrackFormer**	0.809	0.788	0.772	0.819	0.763

## Conclusion

Dynamic video scene classification technology can deeply analyze competition data, providing coaches and athletes with more scientific training guidance and competition strategies. To automatically judge football sports videos and improve the fairness and accuracy of game judgment, this study analyzes the automatic judgment of football sports and proposes a new SODA and scene extraction model. The detection accuracy of the improved YOLOv7 was 0.993, and the floating-point calculation times and detection speed reached 244 flops and 264.245 fps, respectively. The loss function curve converged with the minimum number of iterations and reached a minimum value of 0.09. The maximum association accuracy and detection accuracy of the improved YOLOv7 were 0.958 and 0.926. The highest IOU reached 0.885 and the recall reached 0.961 on the TrackingNet dataset. The BILSTM-SSA model had significant advantages in scene extraction. The highest accuracy was 0.932, F1 was 0.955, and AUC was 0.969. The research method achieved high accuracy and fairness in football judgment, fluctuating between 0.7–0.9. This study promotes the intelligent development of sports and advances the intelligent processing and management of sports video content. However, the scene extraction model designed in the research does not consider network depth, and further research on network layers is necessary for larger detection tasks. Future research work can also refer to the research directions of others to consider using multi-modal deep learning methods to further improve the accuracy and efficiency of scene recognition.

## Supporting information

S1 Minimal data set. (DOCX)

## List of abbreviations:

YOLOv7: You Only Look Once v7
RNN: Recurrent Neural Networks
SODA: Small Object Detection Algorithm
CNN: Convolutional Neural Network
CBS: Convolutional Batch Normalization and Silu Activation Layer
ELAN: Efficient Layer Aggregation Network
MPConv: MaxPool and Convolutional
SPPCSPC: Spatial Pyramid Pooling Convolutional Neural Network
CSP: Cross Stage Partial Connection
SPP: Spatial Pyramid Pooling
PAN: Path Aggregation Network
FPN: Feature Pyramid Network
CBAM: Convolutional Block Attention Module
CAM: Channel Attention Mechanism
SAM: Spatial Attention Mechanism
BiLSTM: Bidirectional Long Short Term Memory
SSA: Sparrow Search Algorithm
SSD: Single Shot Multibox Detector
Faster R-CNN: Faster Region Convolutional Neural Network
DA: Double Attention
ISTIN: Individual Spatial Temporal Inference Network
RHLI: Recurrent Historical Localization Information;
MAP: Mean Average Precision
IOU: Intersection over Union
DBN: Deep Belief Network
MAPE: Mean Absolute Percentage Error
RMSE: Root Mean Square Error
MAE: Mean Absolute Error
ROC: Receiver Operating Characteristic Curve
AUC: Area Under Curve
ViT: Vision Transformers
