# Supplementary material for: Football sports automatic judgment model based on improved YOLOv7 and RNN
Source: PLoS One. 2025 Nov 5;20(11):e0334158. doi: 10.1371/journal.pone.0334158 (PMC12588467; doi:10.1371/journal.pone.0334158)
Supplement: S1 — (DOCX) [file pone.0334158.s001.docx]

The data in Fig 8(a)

| Iterations | 0 | 20 | 40 | 60 | 80 | 100 | 120 |
| --- | --- | --- | --- | --- | --- | --- | --- |
| Improved YOLOv7 | 0.655 | 0.106 | 0.092 | 0.090 | 0.090 | 0.090 | 0.090 |
| DA-ISTIN | 0.673 | 0.260 | 0.201 | 0.202 | 0.201 | 0.202 | 0.202 |
| Improved YOLOv5n | 0.659 | 0.354 | 0.300 | 0.274 | 0.236 | 0.233 | 0.231 |
| RHLI | 0.662 | 0.191 | 0.187 | 0.178 | 0.176 | 0.174 | 0.173 |

The data in Fig 8(b)

| Iterations | 0 | 20 | 40 | 60 | 80 | 100 | 120 |
| --- | --- | --- | --- | --- | --- | --- | --- |
| Improved YOLOv7 | 0.300 | 0.812 | 0.903 | 0.921 | 0.931 | 0.945 | 0.952 |
| DA-ISTIN | 0.300 | 0.732 | 0.814 | 0.833 | 0.839 | 0.848 | 0.853 |
| Improved YOLOv5n | 0.300 | 0.627 | 0.712 | 0.739 | 0.758 | 0.783 | 0.811 |
| RHLI | 0.300 | 0.694 | 0.723 | 0.731 | 0.757 | 0.761 | 0.769 |

The data in Fig 9(a)

| Iterations | 0 | 50 | 100 | 150 | 200 |
| --- | --- | --- | --- | --- | --- |
| Improved YOLOv7 | 0.156 | 0.338 | 0.527 | 0.681 | 0.958 |
| DA-ISTIN | 0.067 | 0.161 | 0.528 | 0.533 | 0.746 |
| Improved YOLOv5n | 0.088 | 0.221 | 0.359 | 0.526 | 0.759 |
| RHLI | 0.097 | 0.245 | 0.339 | 0.482 | 0.784 |

The data in Fig 9(b)

| Iterations | 0 | 50 | 100 | 150 | 200 |
| --- | --- | --- | --- | --- | --- |
| Improved YOLOv7 | 0.101 | 0.402 | 0.524 | 0.639 | 0.926 |
| DA-ISTIN | 0.078 | 0.123 | 0.335 | 0.508 | 0.699 |
| Improved YOLOv5n | 0.119 | 0.278 | 0.357 | 0.536 | 0.770 |
| RHLI | 0.077 | 0.246 | 0.341 | 0.482 | 0.799 |

The data in Fig 10(a)

| Date set | Model | VisDrone2019 | OTB100 | GOT-10k | TrackingNet |
| --- | --- | --- | --- | --- | --- |
| Training set | Improve YOLOv7 | 0.742 | 0.823 | 0.804 | 0.885 |
|  | DA-ISTIN | 0.684 | 0.593 | 0.701 | 0.751 |
|  | RHLI | 0.679 | 0.699 | 0.751 | 0.671 |
|  | Improve YOLOv5n | 0.674 | 0.591 | 0.637 | 0.577 |
| Test set | Improve YOLOv7 | 0.779 | 0.841 | 0.829 | 0.869 |
|  | DA-ISTIN | 0.641 | 0.471 | 0.720 | 0.726 |
|  | RHLI | 0.755 | 0.641 | 0.737 | 0.707 |
|  | Improve YOLOv5n | 0.704 | 0.703 | 0.717 | 0.641 |

The data in Fig 10(b)

| Date set | Model | VisDrone2019 | OTB100 | GOT-10k | TrackingNet |
| --- | --- | --- | --- | --- | --- |
| Training set | Improve YOLOv7 | 0.935 | 0.954 | 0.892 | 0.959 |
|  | DA-ISTIN | 0.843 | 0.820 | 0.815 | 0.855 |
|  | RHLI | 0.836 | 0.822 | 0.814 | 0.816 |
|  | Improve YOLOv5n | 0.858 | 0.824 | 0.818 | 0.796 |
| Test set | Improve YOLOv7 | 0.919 | 0.935 | 0.914 | 0.961 |
|  | DA-ISTIN | 0.812 | 0.832 | 0.810 | 0.867 |
|  | RHLI | 0.820 | 0.887 | 0.794 | 0.803 |
|  | Improve YOLOv5n | 0.843 | 0.793 | 0.848 | 0.794 |

The data in Fig 11(a)

| / | 20 | 40 | 60 | 80 | 100 | 120 |
| --- | --- | --- | --- | --- | --- | --- |
| BILSTM-SSA | 0.276 | 0.270 | 0.263 | 0.202 | 0.217 | 0.103 |
| DBN | 0.321 | 0.382 | 0.367 | 0.353 | 0.346 | 0.333 |
| BILSTM | 0.391 | 0.366 | 0.303 | 0.246 | 0.275 | 0.168 |

The data in Fig 11(b)

| / | 20 | 40 | 60 | 80 | 100 | 120 |
| --- | --- | --- | --- | --- | --- | --- |
| BILSTM-SSA | 0.178 | 0.173 | 0.111 | 0.112 | 0.103 | 0.078 |
| DBN | 0.334 | 0.287 | 0.259 | 0.202 | 0.197 | 0.185 |
| BILSTM | 0.239 | 0.257 | 0.207 | 0.151 | 0.146 | 0.152 |

The data in Fig 11(c)

| / | 20 | 40 | 60 | 80 | 100 | 120 |
| --- | --- | --- | --- | --- | --- | --- |
| BILSTM-SSA | 0.201 | 0.165 | 0.144 | 0.130 | 0.103 | 0.083 |
| DBN | 0.335 | 0.309 | 0.304 | 0.242 | 0.216 | 0.212 |
| BILSTM | 0.242 | 0.231 | 0.193 | 0.193 | 0.154 | 0.126 |

The data in Fig 12(a)

| / | 20 | 40 | 60 | 80 | 100 | 120 |
| --- | --- | --- | --- | --- | --- | --- |
| BILSTM-SSA | 0.105 | 0.242 | 0.337 | 0.496 | 0.761 | 0.933 |
| DBN | 0.099 | 0.216 | 0.322 | 0.398 | 0.614 | 0.775 |
| BILSTM | 0.101 | 0.201 | 0.300 | 0.431 | 0.607 | 0.828 |

The data in Fig 12(b)

| / | 20 | 40 | 60 | 80 | 100 | 120 |
| --- | --- | --- | --- | --- | --- | --- |
| BILSTM-SSA | 0.152 | 0.291 | 0.481 | 0.842 | 0.768 | 0.754 |
| DBN | 0.145 | 0.268 | 0.343 | 0.440 | 0.706 | 0.919 |
| BILSTM | 0.091 | 0.197 | 0.316 | 0.350 | 0.483 | 0.863 |
